# Thermodynamics of the DNA Repair Process by Endonuclease VIII

**Published:** 2019

**Authors:** O. A. Kladova, N. A. Kuznetsov, O. S. Fedorova

**Affiliations:** Institute of Chemical Biology and Fundamental Medicine, Siberian Branch of the Russian Academy of Sciences, Akad. Lavrentiev Ave. 8, 630090, Novosibirsk, Russia; Department of Natural Sciences, Novosibirsk State University, Pirogova Str. 2, 630090, Novosibirsk Russia.

**Keywords:** thermodynamics, pre-steady-state kinetics, kinetic mechanism, DNA glycosylase

## Abstract

In the present work, a thermodynamic analysis of the interaction between endonuclease VIII (Endo VIII) and model DNA substrates containing damaged nucleotides, such as 5,6-dihydrouridine and 2-hydroxymethyl-3-hydroxytetrahydrofuran (F-site), was performed. The changes in the fluorescence intensity of the 1,3-diaza-2-oxophenoxazine (tC°) residue located in the complementary chain opposite to the specific site were recorded in the course of the enzyme-substrate interaction. The kinetics was analyzed by the stopped-flow method at different temperatures. The changes of standard Gibbs free energy, enthalpy, and entropy of sequential steps of DNA substrate binding, as well as activation enthalpy and entropy for the transition complex formation of the catalytic stage, were calculated. The comparison of the kinetic and thermodynamic data characterizing the conformational transitions of enzyme and DNA in the course of their interaction made it possible to specify the nature of the molecular processes occurring at the stages of substrate binding, recognition of the damaged base, and its removal from DNA.

## INTRODUCTION

**Fig. 1:**
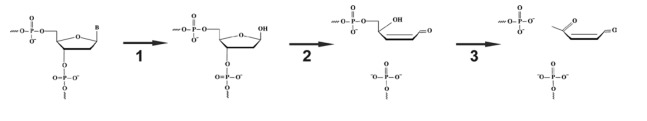
Chemical steps in Endo VIII catalysis. Step 1, DHU base removal; step 2, β-elimination of the 3′-phosphate; step 3, β-elimination of the 5′-phosphate.

Endonuclease VIII (Endo VIII or Nei) is one of the key DNA glycosylases in *Escherichia coli *responsible for the elimination of a wide range of oxidized and reduced pyrimidine bases [1, 2]. The products of oxidation/ reduction of pyrimidine bases in DNA are thymine glycol, 5,6-dihydrothymine, 5,6-dihydrouracil, urea, 5-formyluracil, 5-hydroxymethyluracil, 5-hydroxycytosine, 5-hydroxyuracil, uracil glycol, etc. Endo VIII catalyzes the hydrolysis of the N-glycosidic bond of a damaged base (N-glycosylase activity) with subsequent β-elimination of the 3’ and 5’ phosphate groups of the apurinic/apyrimidinic site (AP lyase activity), resulting in the formation of a single-strand break in DNA (*Fig. 1*) [3, 4].

**Fig. 2:**
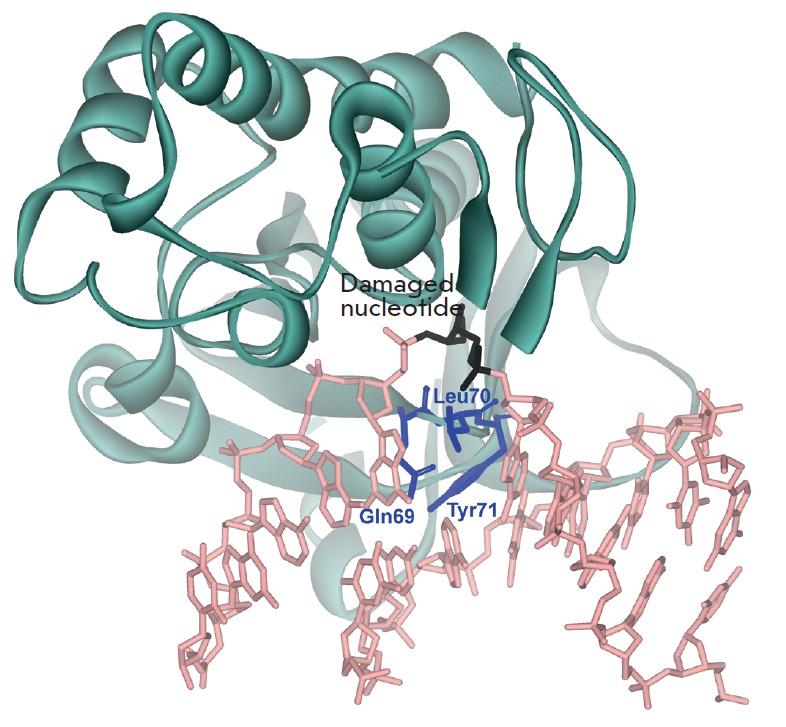
The structure of Endo VIII complexed with DNA containing an AP-site (Protein Data Bank code 1K3W) [6]

X-ray structural analysis of the free enzyme and its complex with DNA showed that the interaction of Endo VIII with DNA leads to conformational changes both in the protein and substrate molecules [5, 6]. In the enzyme-substrate complex, the ribose-phosphate backbone of DNA is bent at approximately 45°, the damaged base is extruded from the DNA helix and inserted in the active center of the enzyme, while the Gln69, Leu70, and Tyr71 residues are positioned into the resulting cavity (*Fig. 2*). Such changes in the structure of interacting molecules lead to the formation of specific contacts, providing highly efficient recognition and binding of damaged DNA nucleotides.

**Scheme 1:**
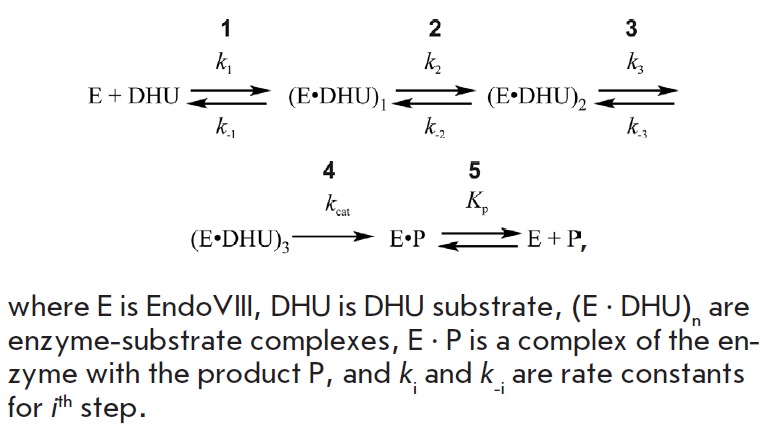
Kinetic mechanism of interaction between Endo VIII and DHU substrate

Previously, we performed the kinetic analysis of conformational changes in Endo VIII and the DNA substrate during their interaction using the stopped-flow method with registration of the changes in the fluorescence intensity of tryptophan residues in the enzyme structure [7] and a number of fluorescent analogues of heterocyclic bases in DNA [8] located on the 3’ side or complementary to the damaged nucleotide. Later [9], we used the strategy of site-directed mutagenesis to clarify the nature of the sequential stages of DNA binding. All the kinetic data characterizing conformational changes in the enzyme and DNA substrates, as well as the results of the mutational analysis, allowed us to propose a molecular kinetic mechanism for the recognition of a damaged nucleotide by the Endo VIII enzyme (*Scheme 1*). Stage 1 corresponds to a rapid initial binding of DNA and formation of a non-specific enzyme-substrate complex in which the N- and C-domains of the enzyme are in a closed position. At this stage, the Leu70 residue is wedged into the DNA duplex, which is the key stage of recognition of the damaged DNA region. Stage 2 involves the bending of the double helix at the damaged base site, the eversion of 5,6-dihydrouracil from the duplex, and the incorporation of the Tyr71 residue in the DNA helix, required for the stabilization of the inverted conformation of the damaged base. In stage 3, the conformation of the active center is adjusted in order to achieve a catalytically competent state. The Tyr71 residue is also utilized in stage 3. In this stage, contacts are formed between Phe121 and the ribose-phosphate backbone of DNA. The formation of the catalytic complex leads to the hydrolysis of the N-glycosidic bond, followed by the β-elimination of the 3’ and 5’ phosphate groups (stage 4). The final stage of the enzymatic cycle is the dissociation of the enzyme-product complex (stage 5).

In order to confirm the kinetic mechanism proposed (*Scheme 1*) and specify the nature of individual stages, we determined the thermodynamic parameters of the fast stages of the interaction between Endo VIII and DNA and the specific recognition of a damaged nucleotide based on the kinetic parameters obtained at different temperatures.

## EXPERIMENTAL

**Oligodeoxyribonucleotides**

Oligonucleotides were purified by HPLC on an ion exchange column (PRP-X500 Hamilton Company 3.9×300 mm; particle size, 12–30 μm), followed by reverse phase chromatography (Nucleoprep 100–20 C18 10×250 mm, Macherey-Nagel, Germany). The purity of the oligonucleotides was verified by denaturing electrophoresis in a 20% polyacrylamide gel (PAAG). The concentration of oligonucleotides was determined based on the optical density of solutions at a wavelength of 260 nm in electronic absorption spectra and molar extinction coefficients calculated using the nearest neighbor method [10].

DNA duplexes of 17 bp containing 1,3-diaza-2-oxophenoxazine fluorophore (tCO), instead of a cytosine residue in the complementary chain opposite the specific site, were used as DNA substrates (*Table 1*). The specific sites were the 5,6-dihydrouridine residue, which served as the damaged base, and an F-site, which is an analog of an intermediate product of the enzymatic reaction; i.e., the apurinic/apyrimidinic site (AP site). A duplex containing no modified nucleotides was used as intact DNA.

**Table 1:** Structure of the DNA-duplexes used in this study

Name	DNA-duplex
DHU-DNA, X = DHU F-DNA, X = F-site G-DNA, X = G	5′-TCTCTCTCXCCTTCCTT-3′ 3′-AGAGAGAG(tCO)GGAAGGAA-5′

**Endonuclease VIII**

Endo VIII was isolated from *E. coli *Rosetta II (DE3) cells transformed with a pET-24b plasmid carrying the enzyme gene. The *E. coli *Rosetta II (DE3) cells were cultivated in a LB medium (1 L) containing 50 μg/ml ampicillin at 37°C until it reached an optical density of 0.6–0.7 at a 600-nm wavelength. After this, the temperature was decreased to 30°C and transcription was induced by addition of isopropyl- β-*D*-thiogalactopyranoside to a final concentration of 0.2 mM. After induction, the cell culture was incubated for 8 h. Next, the cells were precipitated by centrifugation (10 min, 12,000 rpm) and the cell suspension was prepared in 30 ml of a buffer solution (20 mM HEPES-NaOH, pH 7.8; 40 mM NaCl). The cells were lysed using a French press (French Press Cell, Thermo Fisher Scientific, USA). All subsequent procedures were performed at 4°C. The cell lysate was centrifuged (40 min at 30,000 rpm); the supernatant was loaded onto column I (Q-Sepharose Fast Flow, Amersham Biosciences, Sweden) and washed with the buffer solution (20 mM HEPES-NaOH, pH 7.8; 40 mM NaCl). Protein-containing fractions were collected and loaded onto column II (HiTrap-Heparin™, Amersham Biosciences, Sweden). Chromatography was performed in a linear gradient of 40 → 1500 mM NaCl, and the optical density of the solution was measured at 280 nm. The degree of protein purity was determined by gel electrophoresis. Fractions containing the Endo VIII protein were dialyzed in 20 mM HEPES-NaOH buffer, pH 7.5, 1 mM EDTA, 1 mM dithiothreitol, 250 mM NaCl, 50% glycerol and stored at –20°C. Enzyme concentration was calculated based on the optical density of the protein at 280 nm and a molar extinction coefficient of 32680 M^-1^×cm^-1^ [11].

**Kinetic studies using the stopped-flow method**

Kinetic curves were obtained based on the changes in tCO fluorescence intensity on a stopped-flow spectrometer SX.20 (Applied Photophysics, Great Britain). The tCO fluorescence excitation wavelength was 360 nm. Fluorescence was recorded at wavelengths greater than 395 nm (Schott filter GG 395). The instrument dead time was 1.4 ms; the maximum signal acquisition time was 500 s. All experiments were performed in a buffer solution of 50 mM Tris-HCl, pH 7.5, 50 mM KCl, 1 mM dithiothreitol, 1 mM EDTA, 7% glycerol at a temperature range of 5 to 25°C. Each kinetic curve was averaged over at least three experimental curves.

**Analysis of kinetic curves**

In order to calculate the rate constants for conformational transitions, a set of kinetic curves was obtained for different concentrations of the enzyme at different temperatures. The fluorescence intensity of tCO was recorded under conditions close to one enzyme turnover; i.e., at enzyme and substrate concentrations of the same magnitude. To determine the minimum kinetic scheme describing the interaction of the enzyme with a substrate and to calculate rate constants for all elementary reactions corresponding to this scheme, the DynaFit software (BioKin, USA) was used [12]. Quantitative processing of the results was performed by optimization of the values of the parameters comprising the kinetic schemes as previously described [13-16].

Using the values obtained for the rate constants of direct and reverse reactions for individual reversible stages, we calculated the equilibrium constants *K*i for these stages (*K*i = *k*i/*k*-i, where *i *is the stage number) at different temperatures. The standard thermodynamic parameters of the *i*th equilibrium stage were determined using the Van’t Hoff equation (1) [17, 18] as previously described [19-23].

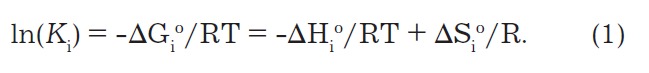



Analysis of the temperature dependence of the *k*cat rate constant using the Eyring equation (2) allowed us to calculate the standard activation enthalpy (ΔHo,‡) and the standard activation entropy (ΔSo,‡) of the transition state [17].

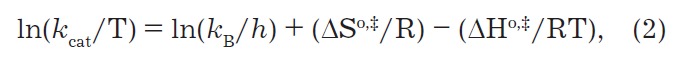



where *k*B and *h *are the Boltzmann and Planck constants, respectively; R is the universal gas constant; T is the absolute temperature in Kelvin degrees; and *k*_cat_ is the rate constant for the irreversible catalytic stage.

## RESULTS AND DISCUSSION

**Interaction between Endo VIII and intact G-DNA**

**Fig. 3:**
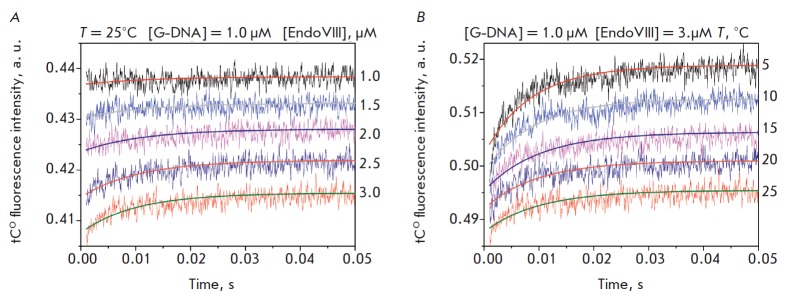
Interaction of Endo VIII with intact G-DNA. *A *– Changes in the tCO fluorescence intensity. Jagged traces represent experimental data; smooth curves are the results of fitting to *Scheme 2*. [G-DNA] = 1.0 μM, concentrations of Endo VIII (1.0−3.0 μM) are shown on the right side of the plot. *B *– Changes in the tC° fluorescence intensity of the interaction of Endo VIII with G-DNA at different temperatures ([Endo VIII] = 3.0μM, [G-DNA] = 1.0μM)

**Scheme 2:**
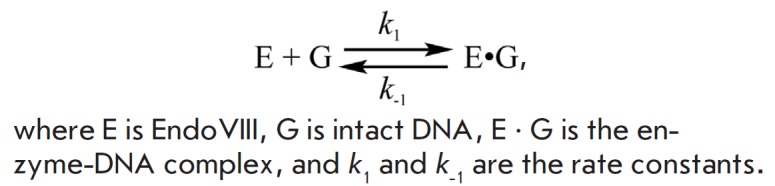
Kinetic mechanism of interaction between Endo VIII and intact DNA

*Figure 3A* presents the kinetic curves for the interaction between Endo VIII and the DNA duplex G-DNA containing a guanine residue complementary to the fluorophore group tCO obtained by registration of the changes in the tCO fluorescence intensity during the reaction. One phase of growth in the fluorescence intensity, which reaches a plateau, can be noted on the kinetic curves. When increasing the temperature, the time necessary to reach the plateau shifts from ~30 ms at 5°C to ~20 ms at 25°C (*Fig. 3C*). The kinetic curves obtained are satisfactorily described by single-stage equilibrium kinetic *Scheme 2*.

**Table 2:** Rate and equilibrium constants of the interaction of Endo VIII with intact G-DNA

Constants	Temperature, °C				
	5	10	15	20	25
k_1_, M^-1^s^-1^	(16±7)×10^6^	(16±8)×10^6^	(15±9)×10^6^	(14±6)×10^6^	(9±2)×10^6^
k^-1^, s^-1^	50±30	60±40	50±20	60±40	40±10
K^1^, M^-1^	(0.31±0.21)×10^6^	(0.26±0.21)×10^6^	(0.29±0.20)×10^6^	(0.25±0.18)×10^6^	(0.19±0.08)×10^6^

Scheme 2

**Interaction of Endo VIII with AP site analog F-DNA**

**Fig. 4:**
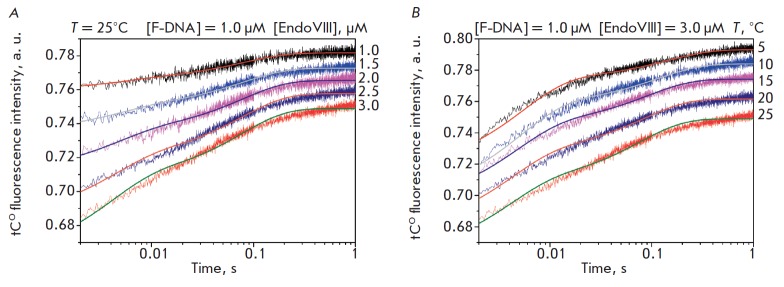
Interaction of Endo VIII with F-DNA. *A *– Changes in the tCO fluorescence intensity. Jagged traces represent experimental data; smooth curves are the results of fitting to *Scheme 3*. [F-DNA] = 1.0 μM, concentrations of Endo VIII (1.0−3.0 μM) are shown on the right side of the plot. *B *– Changes in the tCO fluorescence intensity of the interaction of Endo VIII with F-DNA at different temperatures ([Endo VIII] = 3.0μM, [F-DNA] = 1.0μM)

Interaction between Endo VIII and the AP site in DNA has been studied using a DNA duplex containing a noncleavable analog of the AP site (tetrahydrofuran F derivative) and the fluorophore group tCO opposite a damaged nucleotide in the complementary chain (*Fig. 4*). Two phases of tCO fluorescence intensity growth can be distinguished on the kinetic curves presented in *Fig. 4*. The first phase of growth takes place in the same time range (up to ~20 ms) as in the case of interaction with intact G-DNA. The same conformational transformation caused by binding to Endo VIII is likely to occur in the structure of DNA duplexes containing both the G and F sites at the initial time. However, there is a second stage of tCO fluorescence intensity growth for the F ligand which is completed by the 1^st^ second at all temperatures (*Fig. 4*). In addition, the changes in the kinetic curves of the interaction between an F-containing DNA and Endo VIII have a higher amplitude than in the case of G-DNA, suggesting both greater efficiency of complex formation between the enzyme and DNA and more significant conformational rearrangements in the structure of the DNA duplex containing the F-site.

**Scheme 3:**
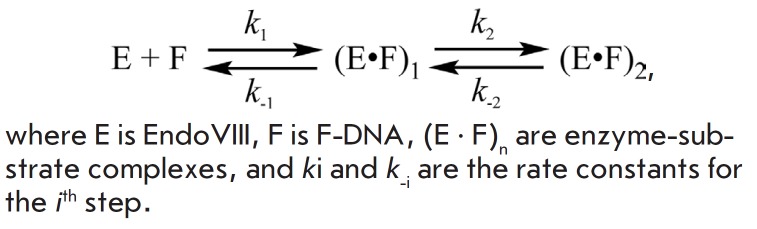
Kinetic mechanism of interaction between Endo VIII and F-DNA

Processing of the data obtained resulted in the determination of a minimal kinetic reaction scheme which included two reversible stages of the formation of the enzyme-substrate complex (*Scheme 3*). The reaction rate constants and equilibrium constants calculated are presented in *Table 3*.

**Table 3:** Rate and equilibrium constants of the interaction of Endo VIII with F-DNA

Constants	Temperature, °C				
	5	10	15	20	25
k_1_, M^-1^s^-1^	(35±9)×10^6^	(35±5)×10^6^	(37±5)×10^6^	(39±5)×10^6^	(40±8)×10^6^
K_-1_, M^-1^	89±22	99±18	109±28	129±43	144±46
K_1_, M^-1^	(0.4±0.1)×10^6^	(0.35±0.08)×10^6^	(0.3±0.1)×10^6^	(0.3±0.1)×10^6^	(0.3±0.1)×10^6^
k_2_, s^-1^	0.3±0.1	0.9±0.3	1.4±0.2	3±1	3±1
k_-2_, s^-1^	7±2	8±1	10±1	11±1	12±1
K_2_	0.04±0.02	0.11±0.03	0.13±0.02	0.24±0.09	0.26±0.08

Scheme 3

**Interaction of Endo VIII with DHU-DNA**

**Fig. 5:**
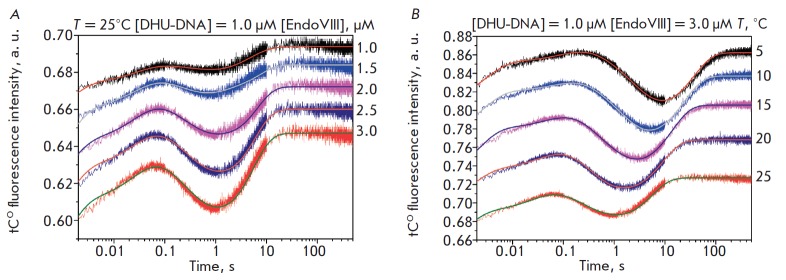
Interaction of Endo VIII with DHU-DNA. *A *– Changes in the tCO fluorescence intensity. Jagged traces represent experimental data; smooth curves are the results of fitting to *Scheme 1*. [DHU-DNA] = 1.0 μM, concentrations of Endo VIII (1.0-3.0 μM) are shown on the right side of the plot. *B *– Changes in the tCO fluorescence intensity of the interaction of Endo VIII with DHU-DNA at different temperatures ([Endo VIII] = 3.0μM, [DHU-DNA] = 1.0μM)

*Figure 5A* presents the kinetic curves obtained for the interaction between Endo VIII and the DNA substrate containing 5,6-dihydrouridine (DHU-DNA). The curves are even more complicated than those obtained for G-DNA and F-DNA. Similar changes in the tCO fluorescence intensity can be noted for all concentration series at different temperatures (5–25°C) (*Fig. 5B*).

Analysis of the kinetic curves registered at 5–15°C demonstrated a rapid growth in the tCO fluorescence intensity at the initial region (up to 10 ms) (phase 1). At increased temperatures, this change in the fluorescence intensity disappears almost completely. Following the 1^st^ growth phase, further increase in the fluorescence signal can be identified at all temperatures. The duration of the 2^md^ phase of growth decreased with an increase in the temperature from ~300 ms at 5°C to ~80 ms at 25°C.

It is known that the changes in the DNA helix structure, the extrusion of the damaged nucleotide, and the incorporation of a series of amino acid residues of the enzyme into the DNA helix occur when Endo VII binds to DNA [6]. Endo VIII forms an extensive network of contacts with DNA. However, the contacts formed by the amino acid residues of the triad Gln69–Leu70– Tyr71 play the most important role in the recognition of a damaged nucleotide.

**Table 4:** Rate and equilibrium constants of the interaction of Endo VIII with DHU-DNA

Constants	Temperature, °C				
	5	10	15	20	25
k_1_, M^-1^s^-1^	35±7	40±6	45±7	61±4	80±13
k_-1_, s^-1^	230±30	270±40	320±10	440±70	580±50
K_1_, M^-1^	(0.149±0.038)×10^6^	(0.147±0.032)×10^6^	(0.140±0.023)×10^6^	(0.140±0.025)×10^6^	(0.138±0.027)×10^6^
k_2_, s^-1^	1.0±0.2	1.7±0.1	2.6±0.4	3.6±0.6	4±1
k_-2_, s^-1^	0.34±0.08	0.62±0.03	0.9±0.7	1.2±1	1.3±0.3
K_2_	2.72±0.94	2.76±0.24	2.83±2	3.0±2.7	3.0±1
k_3_, s^-1^	6.5±1.4	8.1±0.6	12±2	18±4	29±3
k_-3_, s^-1^	1.6±0.3	1.9±0.6	2.5±0.6	3±1	4.4±1.6
K_3_	4±1	4.3±1.4	4.6±1.3	5.3±1.9	6.6±2.5
k_cat_, s^-1^	0.06±0.02	0.09±0.05	0.15±0.05	0.22±0.08	0.34±0.02
K_5_, M^-1^	(0.06±0.03)×10^6^	(0.047±0.019)×10^6^	(0.042±0.016)×10^6^	(0.038±0.010)×10^6^	(0.034±0.18)×10^6^

For all the curves obtained, one can distinguish the phase of drop in the tCO fluorescence intensity (*phase 3*). This phase has a pronounced temperature dependence. For instance, the phase of drop in the tCO fluo rescence intensity lasts up to 10 s at 5°C and only up to 1 s at 25°C. Such a change corresponds to another stage occurring during the formation of the enzyme-substrate complex. Since a decrease in the tCO fluorescence intensity indicates a change in the microenvironment of the fluorophore group, one can assume the adjustment of the enzyme and DNA conformations to take place at this moment to form a catalytically competent enzyme-substrate complex.

An increase in the fluorescence signal (phase 4), followed by the reaching of a plateau, (phase 5) is observed immediately after a drop in tCO fluorescence intensity. It is most likely that the fourth stage of change in the fluorescence intensity reflects the catalytic stages in the enzymatic process, while the fifth stage represents the dissociation of the enzyme complex from the reaction product.

Thus, a total of five phases of changes in the tCO fluorescence intensity have been identified in the kinetic curves of interaction between endonuclease VII and a DNA substrate containing 5,6-dihydrouridine. A minimal kinetic scheme describing the kinetic curves includes three reversible stages that lead to the formation of an enzyme-substrate complex, one irreversible stage, which can be attributed to the catalytic reaction stage, and one reversible stage of the enzyme-product complex dissociation (*Scheme 1*). The calculated values of the rate constants for individual stages and equilibrium constants are presented in *Table 4*.

**Fig. 6:**
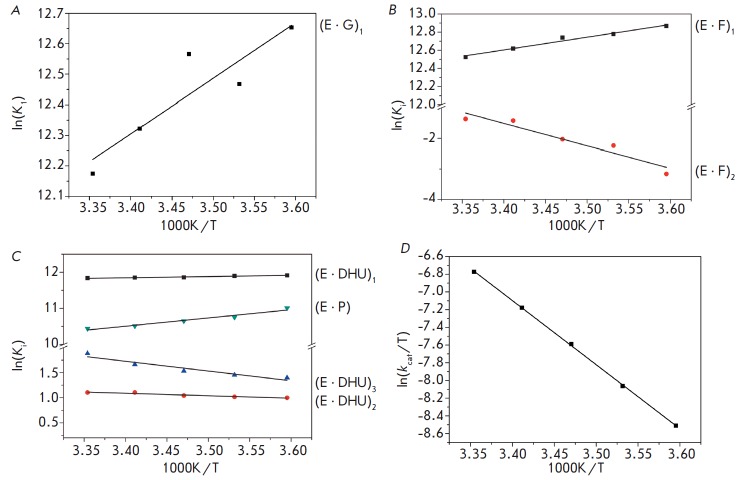
Van’t Hoff analysis of the temperature dependence of *K*i for G-DNA (*A*), F-DNA (*B*), and DHU-DNA (*C*). (*D*) Analysis of the temperature dependence of ln(*k*cat/T) characterizing the transition state of the catalytic step for DHU-DNA

**Thermodynamic parameters of the interaction between Endo VIII and DNA substrates**

The rate constants for the individual stages of the interaction between Endo VIII and all DNA substrates at different temperatures were used to calculate equilibrium constants for these stages (*K*_i_). The equilibrium constants of individual stages were used for obtaining the dependence of ln(*K*i) on 1/T (van’t Hoff equation (1)) (*Fig. 6*). The dependence of ln(*k*^cat^/T) on 1/T (Eyring equation (2)) that characterizes the irreversible catalytic reaction in the case of the DHU substrate was also obtained. All dependencies had a linear form and allowed us to calculate the changes in enthalpy and entropy for reversible stages (ΔH_i_^o^ and ΔS_i_^o^) and the transition state of the catalytic stage (ΔH^o,‡^ and ΔS^o,‡^) (*Table 5*).

**Table 5:** Thermodynamic parameters of interaction of Endo VIII with DNA

DNA substrate	Stage No	ΔH_i_°, kcal/mol	ΔS_i_°, cal/(mol×K)	ΔG^o^_i,298_, kcal/mol	Stage description^a^
G/tC°	1	-3.6 ± 0.9	12 ± 3	-7.2	Primary binding, attempt at incorporation of Leu70 residue, increase in the polarity of the tC° environment
F/tC°	1	-2.8 ± 0.3	16 ± 1	-7.4	Primary binding, intercalation of Leu70, increase in the polarity of the tC° environment
	2	15 ± 3	47 ± 9	0.8	Bending of the double helix, increase in the polarity of the tC° environment
	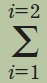	12.2 ± 3.3	60 ± 10	-6.6	
DHU/tC°	1	-0.7 ± 0.1	21.3 ± 0.4	-7.0	Primary binding, increase in the polarity of the tC° environment
	2	1.0 ± 0.2	5.5 ± 0.6	-0.65	Bending of the double helix, increase in the polarity of the tC° environment
	3	3.9 ± 0.7	17 ± 2	-1.1	Formation of a catalytic complex, decrease in the polarity of the tC° environment
	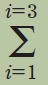	4.2	43.8	-8.75	
	4^b^	14.4 ± 0.1	-12.4 ± 0.5	18	Catalysis, increase in the polarity of the tC° environment
	5	-4.5 ± 0.6	5 ± 2	-6.2	Formation of a complex with the reaction product, increase in the polarity of the tC° environment

^a^aAccording to [7–9].

^b^Parameters calculated using the Eyring equation (2).

When analyzing the thermodynamic parameters of the interaction between Endo VIII and DNA substrates, we succeeded in identifying some common features. For instance, primary binding of the enzyme to all the DNA substrates used in the study is accompanied by a slight decrease in enthalpy and an increase in entropy. It leads to a negative value of the change in the Gibbs energy ΔGo1 for the first stage of the formation of the enzyme-substrate complex. It is worth noting that the value of ΔGo1,298 (ranging from -7.0 to -7.4 kcal/mol) is similar for both damaged and intact DNA, indicating that the interaction of the enzyme with DNA is energetically favorable.

The thermodynamic parameters of the first stage obtained for G-DNA, F-DNA, and DHU-DNA indicate that the initial stage of interaction (up to 10 ms) between Endo VIII and DNA is identical for all of the DNA duplexes. No specific contacts with the damaged nucleotide take place at the stage of primary binding. The recognition of the damaged nucleotide occurs later, upon its eversion from the DNA helix and incorporation of the Gln69–Leu70–Tyr71 triad into the helix. Nevertheless, it has been established [9] that Endo VIII utilizes Leu70 as a “sensor” of damaged nucleotides and that its intercalation into the duplex structure takes place at early stages of specific enzyme-substrate interaction. Therefore, one can assume that an increase in the tCO fluorescence intensity at the initial regions of all kinetic curves reflects local conformational changes in the DNA duplex upon wedging of the Leu70 residue into the DNA helix.

The thermodynamic analysis of the second stage of recruitment of Endo VIII to DNA duplexes observed in the case of F- and DHU-containing duplexes revealed a degree of difference. A positive ΔG^o^_2,298_ value in the case of F-DNA indicates that the process is unfavorable and does not appear to take place at low temperatures. In the case of the DHU-containing substrate, the second stage of enzyme-substrate complex formation is energetically neutral; the ΔG^o^_2,298_ value is equal to -0.65 kcal/mol. This stage is accompanied by an increase in ΔH^o^ and ΔS^o^ for both DNA duplexes. According to previously obtained data, at this stage, duplex bending occurs, which should be accompanied by the insertion of the damaged nucleotide into the active center of the enzyme and complete incorporation of all residues of the Gln69–Leu70–Tyr71 triad into the DNA helix [8].

Final adjustment of the active center structure occurs for the transition to the catalytic stage at the third stage of interaction between Endo VIII and the DHU substrate, which precedes the catalytic reaction. The significant increase in entropy observed at this stage is most likely due to the dissolving of polar groups in the region of the enzyme–DNA contact, as well as the removal of water molecules from the grooves of the DNA substrate. A positive change in the enthalpy ΔH^o^ indicates energy costs in obtaining a catalytically active conformation. This is followed by the irreversible catalytic stage (stage 4), which includes hydrolysis of the N-glycosidic bond of the damaged nucleotide and formation of a gap in the sugar-phosphate backbone of the DNA at the 3’- and 5’-sides from the damaged nucleotide. The catalytic stage is accompanied by a large expenditure of energy, as indicated by the positive values of ΔG^o,‡^_298_ = 18.0 kcal/mol and ΔH^o,‡^ = 14.4 kcal/mol. The final stage of interaction between Endo VIII and a DHU-containing substrate is the dissociation of the enzyme complex from the product. It should be noted that ΔG^o^_298_ (-6.2 kcal/mol) of this stage has a value similar to that of ΔG^o^_298_ for the primary DNA binding stage (-7.0 to -7.4 kcal/mol).

## CONCLUSION

The changes in the fluorescence intensity of 1,3-diaza- 2-oxophenoxazine in the X:tCO pair (X = G, F, DHU) were recorded for all of the Endo VIII DNA glycosylase substrates used in the study. It has been shown that the first phase of tCO fluorescence intensity growth is characteristic of the kinetic curves obtained for all of the substrates. According to the thermodynamic parameters obtained, this stage reflects a similar stage of the primary binding of Endo VIII to DNA. According to [7, 8, 9], this stage presents closure of the enzyme domains and an attempt to incorporate Leu70 into the DNA helix. The second phase of growth in fluorescence intensity is recorded only for DNA duplexes carrying the damaged nucleotide F or DHU. This change corresponds to the stage of second enzyme-substrate complex formation. The insertion of the damaged nucleotide into the active center of Endo VIII and incorporation of the amino acid residues of Endo VIII into the DNA double helix is likely to occur at this stage. For DHU-DNA, this stage lasts up to 1 s. It is worth noting that both enthalpy and entropy increase in this case. However, the change in the Gibbs energy ΔGoi,298 is close to zero (0.8 and -0.65 kcal/mol for F-DNA and DHU-DNA, respectively) at this stage. Hence, the energy costs of changing the structure of the enzyme and DNA substrate molecules are compensated by the increase in the entropy of the system. The kinetic curves (*Fig. 5*) obtained for the DHU-containing duplex show that the two phases of growth in the tCO fluorescence intensity are followed by a phase of decrease, reflecting the formation of the third enzyme-substrate complex. The final verification of the structure of the damaged nucleotide and formation of a catalytically active complex is carried out at this stage. In this case, the fluorescence intensity of tCO is minimal, indicating the formation of the most hydrophobic environment for the fluorophore group. Also, this stage is accompanied by an increase in entropy, probably indicating the removal of water molecules from the enzyme-substrate contact region and, consequently, compaction of the enzyme– substrate complex. The thermodynamic parameter values of the fast stages of interaction between Endo VIII and DNA are consistent with previously obtained data on the mechanism of recognition and conversion of the specific site by the enzyme [7, 8, 9]. The relative changes in the thermodynamic parameters of individual fast stages of the enzymatic process catalyzed by Endo VIII DNA glycosylase are in agreement with the values previously obtained by us for other DNA glycosylases, such as Fpg [19], hOGG1 [20], and Nth [22].

## Abbreviations

Endo VIII: endonuclease VIII
AP-site: apurinic/apyrimidinic site
F-site: (3-hydroxytetrahydrofuran-2-yl) methyl phosphate
DHU: 5,6-dihydrouridine
